# Collective Dynamics Differentiates Functional Divergence in Protein Evolution

**DOI:** 10.1371/journal.pcbi.1002428

**Published:** 2012-03-29

**Authors:** Tyler J. Glembo, Daniel W. Farrell, Z. Nevin Gerek, M. F. Thorpe, S. Banu Ozkan

**Affiliations:** 1 Center for Biological Physics, Department of Physics, Arizona State University, Tempe, Arizona, United States of America; 2 Laufer Center for Physical and Quantitative Biology, Stony Brook University, Stony Brook, New York, United States of America; National Cancer Institute, United States of America and Tel Aviv University, Israel, United States of America

## Abstract

Protein evolution is most commonly studied by analyzing related protein sequences and generating ancestral sequences through Bayesian and Maximum Likelihood methods, and/or by resurrecting ancestral proteins in the lab and performing ligand binding studies to determine function. Structural and dynamic evolution have largely been left out of molecular evolution studies. Here we incorporate both structure and dynamics to elucidate the molecular principles behind the divergence in the evolutionary path of the steroid receptor proteins. We determine the likely structure of three evolutionarily diverged ancestral steroid receptor proteins using the Zipping and Assembly Method with FRODA (ZAMF). Our predictions are within ∼2.7 Å all-atom RMSD of the respective crystal structures of the ancestral steroid receptors. Beyond static structure prediction, a particular feature of ZAMF is that it generates protein dynamics information. We investigate the differences in conformational dynamics of diverged proteins by obtaining the most collective motion through essential dynamics. Strikingly, our analysis shows that evolutionarily diverged proteins of the same family do not share the same dynamic subspace, while those sharing the same function are simultaneously clustered together and distant from those, that have functionally diverged. Dynamic analysis also enables those mutations that most affect dynamics to be identified. It correctly predicts all mutations (functional and permissive) necessary to evolve new function and ∼60% of permissive mutations necessary to recover ancestral function.

## Author Summary

Proteins are remarkable machines of the living systems that show diverse biochemical functions. Biochemical diversity has grown over time via molecular evolution. In order to understand how diversity arose, it is fundamental to understand how the earliest proteins evolved and served as templates for the present diverse proteome. The one sequence - one structure - one function paradigm is being extended to a new view: an ensemble of different conformations in equilibrium can evolve new function and the analysis of inherent structural dynamics is crucial to give a more complete understanding of protein evolution. Therefore, we aim to bring structural dynamics into protein evolution through our zipping and assembly method with FRODA. (ZAMF). We apply ZAMF to simultaneously obtain structures and structural dynamics of three ancestral sequences of steroid receptor proteins. By comparative dynamics analysis among the three ancestral steroid hormone receptors: (i) we show that changes in the structural dynamics indicates functional divergence and (ii) we identify all functionally critical and most of the permissive mutations necessary to evolve new function. Overall, all these findings suggest that conformational dynamics may play an important role where new functions evolve through novel molecular interactions.

## Introduction

Proteins are effective and efficient machines that carry out a wide range of essential biochemical functions in the cell. Beyond being robust and efficient, the outstanding property of proteins is that they can evolve and they show a remarkable capacity to acquire new functions and structures. In fact, modern proteins have emerged from only a few common ancestors over millions to billions of years [1]–[3]. Moreover, the emergence of drug resistance and enzymes with the capacity to degrade new chemicals indicates the ongoing contemporary evolution of proteins [1]–[7]. Therefore, understanding the mechanism by which mutations lead to functional diversity is critical in many aspects from protein engineering to drug design and personalized medicine. Indeed, computational protein design through analysis of mutations has attained major breakthroughs, with profound biotechnological and biomedical implications: design of a new fold [8], design of new biocatalysts and biosensors [9]–[11], design of binding affinity [12], [13], and design of proteins to bind non-biological cofactors [14]. Moreover, there are computational bioinformatics-based tools based on evolutionary information aspects to identify mutations leading to functional loss or disease [15]–[17].

From a phylogenetics perspective, horizontal and vertical approaches have been used to analyze the set of mutations that lead to changes in protein function throughout evolution [18]. The horizontal approach compares modern day proteins at the tips of the evolutionary tree. It identifies the amino acid residue differences within the functionally divergent members of a protein family based on primary sequence and structural analyses and then characterizes the functional role of these residues by swapping them between these family members through site-directed mutagenesis in the laboratory to check for loss of function [19]–[21]. Although the horizontal method gives insight into mutations critical to function, it often fails to identify permissive mutations necessary to switch function between family members. Protein function has evolved as mutations throughout history, i.e. *“vertically”*, in the ancestral protein lineages. Therefore, it is important to incorporate the historical background which contains both neutral and key function-switching mutations when examining function-altering mutations [18]. The vertical approach determines the likely ancestral sequences at nodes along the evolutionary tree and compares modern day proteins to their ancestors. Recent advances in molecular phylogenetic methods make it possible to obtain ancestral sequences by protein sequence alignments in a phylogenetic framework using Bayesian and Maximum Likelihood methods [22], [23]. DNA molecules are synthesized coding for the most probable ancestral sequences and the protein expressed, allowing for experimental characterization of the ancient protein. The vertical approach has been used to gain insight into the underlying principles of protein function and evolution in several proteins including opsins [24], [25], GFP-like protein [26], [27], and others [28]–[32]. More recently, a vertical analysis of two ancestral nuclear receptors has been coupled with X-ray structure determination in successfully elucidating the switching of function between divergent members [33], [34]. Such studies highlight the importance of including ancient protein structures into evolutionary studies.

Although coarse-grained and all-atom models have furthered our understanding of sequence/structure relationship in evolution, further study of the inherent structural dynamics is crucial to give a more complete understanding of protein evolution [35]. A small local structural change due to a single mutation can lead to a large difference in conformational dynamics, even at quite distant residues due to structural allostery [36]–[38]. Thus the one sequence-one structure-one function paradigm is being extended to a new view: an ensemble of different conformations in equilibrium that can evolve new function [1], [39]–[41]. The importance of structural dynamics has been demonstrated by a recent experimental study which shows that mutations distant from a binding site can increase enzyme efficiency by changing the conformational dynamics [42]. The modulation of rigidity/flexibility of residues both near and distant from the active region(s) as related to promiscuous and specific binding has also been noted in tRNA synthetase complexes [43], [44].

Here we have developed a method to predict structural and dynamic evolution of ancestral sequences by using a modified version of our protein structure prediction tool, Zipping and Assembly Method with FRODA (ZAMF) [45]. ZAMF combines two crucial features of ZAM [46], and FRODA [47], [48] : i) FRODA is a constraint-based geometric simulation technique that speeds up the search for native like topologies by accounting only for geometric relationships between atoms instead of detailed energetics, ii) Molecular dynamics identifies the low free energy structures and further refines these structures toward the actual native conformation. Thus, it is a two-step multi-scale computational method that performs fast and extensive conformational sampling. As an outcome, we not only predict protein structures but also obtain detailed conformational dynamics of the predicted structures.

With modified ZAMF, we analyze the role of structural dynamics in the evolution of three ancestral steroid receptors (AncCR, AncGR1 and AncGR2), the ancestors of mineralocorticoid and glucocorticoid receptors (MR and GR). MR and GR arose by duplication of a single ancestor (AncCR) deep in the vertebrate lineage and then diverged function. MR is activated by aldosterone to control electrolyte homeostasis, kidney and colon function and other processes [33]. It is also activated by cortisol, albeit to a lesser extent [18]. On the other hand, GR regulates the stress response and is activated only by cortisol [33]. The structural comparison of human MR and GR (i.e. horizontal approach) suggested the two mutations (S106P and L111Q) to be critical in ligand specificity, however, swapping these residues between human MR and human GR yielded receptors with no binding activity [49]. Conversely, by resurrecting key ancestral proteins (AncCR, AncGR1 and AncGR2) in MR and GR evolution and determining the crystal structures, Thornton *et al.* were able to shed insight into how function diverges through time by using both functional and permissive (compensatory) mutations [33], [34]. AncCR (main ancestor), ∼470 million years old, is a promiscuous steroid receptor which is activated by aldosterone, cortisol, and deoxycortisol ligands. AncCR branched into the mineralocorticoid steroid receptors. AncGR1 (ancestor of sharks) is ∼440 million years old with 25 mutations from AncCR and also promiscuously binds to and functions with aldosterone, cortisol, and deoxycortisol. AncGR1 later evolved into the Elasmobranch glucocorticoid receptor protein. AncGR2 (ancestor of humans and fish) is ∼420 million years old with 36 mutations from AncGR1 and preferentially binds to cortisol alone. These two ancestral proteins, AncGR1 and AncGR2, which diverge functionally, have highly similar experimental structures that have <1 Å RMSD between them. Among 36 mutations between AncGR1 and AncGR2, two conserved mutations {S106P, L111Q} (i.e. group X) when introduced together are sufficient to increase cortisol specificity. However three more functionally critical conserved mutations {L29M, F98I, S212Δ} (i.e. group Y) are needed for the loss of aldosterone binding activity when they are introduced together with two other permissive (i.e. compensatory) mutations {N26T and Q105L} (i.e. group Z). Thus, making the X, Y, Z mutations in AncGR1 enables AncGR1 to function as AncGR2 (i.e. forward evolution) [34]. To make AncGR2 function as AncGR1 (backward evolution) the X, Y, Z mutations are insufficient and render the protein inactive. A fourth set of permissive mutations (W) is required to reverse function in addition to the X, Y, and Z, sets. The W mutation set is {H84Q, Y91C, A107Y, G114Q, L197M} [33]. A mutation between AncCR and AncGR1, Y27R, is also a necessary mutation to eventually alter function to cortisol specificity, though it was not experimentally considered as part of the X, Y, Z, or W mutation sets [34].

We ask here whether an analysis of the predicted 3-D structures and corresponding equilibrated dynamics can distinguish the functional divergence and function swapping mutations between AncCR, AncGR1, and AncGR2. By applying ZAMF, we obtain the 3-D structures within ∼2.7 Å all-atom RMSD of the experimental structures. More importantly, when we analyze their structure-encoded dynamics, we observe that changes in the dynamics indicate functional divergence: that the most collective fluctuation profiles of AncCR and AncGR1 (i.e. the slowest mode) are much closer and distinctively separated from the functionally divergent AncGR2. Moreover, AncCR and AncGR1 have a more flexible binding pocket, suggesting the role of flexibility in their promiscuous binding specificity. On the other hand, the mutations of AncGR2 lead to a rigid binding pocket, which suggests that as the binding becomes cortisol specific, evolution acts to shape the binding pocket toward a specific ligand. Finally, using their mean square fluctuation profiles and cross correlation maps to analyze the change in dynamics at each residue position enables us to distinguish critical mutations needed for swapping the function. Overall, all these findings suggest that conformational epistasis may play an important role where new functions evolve through novel molecular interactions and an analysis of detailed dynamics might provide insight into the mechanisms behind these novel interactions.

## Results/Discussion

### Structure Prediction and Identification of Function Altering Mutations through Structural Analysis

**Figure 1:**
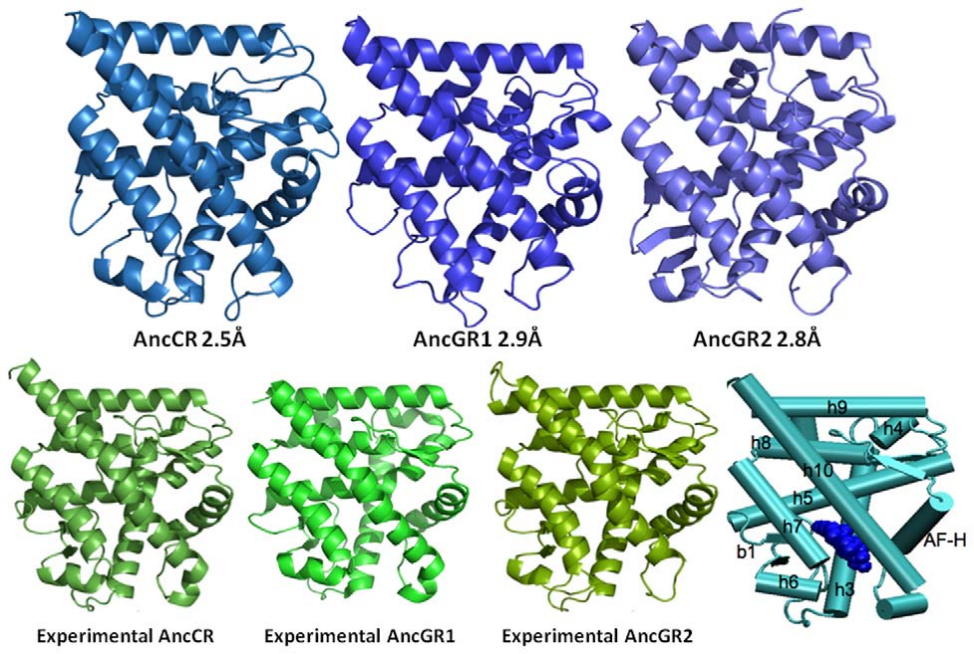
3D structures of AncCR, AncGR1 and AncGR2. AncCR was within 2.5 Å all-atom RMSD from the experimentally determined AncCR. AncGR1 was within 2.9 Å all-atom RMSD from the experimentally determined crystal structure. AncGR2 was within 2.8 Å all-atom RMSD from the experimentally determined AncGR2. Included for reference is a cartoon figure with helices labeled for reference and the ligand is bound, represented in blue spheres.

Many of the modern day homologs to ancestral proteins in the steroid receptor class of the nuclear receptor superfamily have high sequence similarity (∼40–50%), and, as prediction accuracy scales with sequence similarity [50]–[52] our secondary structures for the ancestral sequences are sufficiently accurate to provide native-like structures [45]. Indeed, predicted secondary structures are all correct within one residue to the experimentally determined ancestral cortisol receptor protein [34]. Using these secondary structures as input to the assembly and refinement stages of ZAMF, we determine the 3D structure of the AncCR from its experimentally determined structure to 2.5 Å all atom RMSD (2.2 Å backbone), AncGR1 from its experimentally determined structure to 2.9 Å all atom RMSD (2.6 Å backbone) AncGR2 from its experimentally determined structure to 2.9 Å all atom RMSD (2.4 Å backbone) (Fig. 1 and Table S1). To test the accuracy of these predictions, we first compare the structural differences between the experimental structures. The experimental structures are very similar, with an RMSD of 1.49 Å between AncCR and AncGR1, 1.68 Å between AncCR and AndGR2, and 1.70 Å between AncGR1 and AncGR2. However alignment excludes the atoms of the mutational residues. We also ran a 4 ns REMD simulation of the experimentally determined AncCR and AncGR2 under the same conditions. The ensembles for AncCR and AncGR2 converges at ∼2.5 Å backbone RMSD from their respective experimentally determined structures (Fig. S1). The 2.5 Å RMSD indicates that our predicted structures are as accurate as our force field permits. Closer analysis reveals that helix h9 in the predicted structure of AncGR2 is slightly less stable than in the experimental structure REMD simulations. However, both simulations show a high degree of flexibility in the loop region between helices h9 and h10 and ends of helices h9 and h10 at this loop region.

As these three proteins diverged in function and have >10% sequence mutation between each successive protein, we expect to see some differences in structure. Therefore, we first look at a mean square displacement (MSD) between the static structures of AncCR, AncGR1 and AncGR2. The MSD versus residue profile gives an indication of which residues are mutating, as mutated residues pack into stereochemically unique conformations (Fig. S2). Fig. S2 reveals conformational shifts in helices h7 and h10 and in the β-sheet region, b1. We attempt to determine which of the 36 mutated residues between AncGR1 and AncGR2 are critical for cortisol binding specificity through distinguishing residues having an MSD cutoff of >6 Å^2^ between the AncGR1 and AncGR2 predicted structures. The residues identified from X, Y, Z and W sets are Y91C, Q105L, and S212Δ, with no false positives. The S212Δ and Q105L mutations are permissive mutations to shift function to cortisol specificity whereas Y91C is a permissive mutation necessary for “reverse evolution” i.e. to return binding promiscuity to AncGR2. Experimental work indicates that S212Δ removes a hydrogen bond and imparts greater mobility to the loop before the activation function (AF) helix, allowing it to hydrogen bond with helix h3, while Q105L indirectly restores a hydrogen bond with the activation helix by allowing for tighter packing of helices h3 and h7 [34]. An analysis of hydrogen bonding patterns [53] shows the loss of the S212 hydrogen bond with V217 (in the loop before the AF helix) in the AncGR2 structure as compared to the AncCR/AncGR1 structures, agreeing with experimental results. Y91C is one of the W mutations required for reverse evolution of AncGR1 from AncGR2 and we find it forms a hydrogen bond with N86 in AncGR2 but does not in AncCR or AncGR1. Interestingly, none of these mutations occur in the binding pocket itself. Therefore, an MSD analysis is not sensitive enough to find functionally critical mutations in the binding pocket, and only finds a few of the necessary mutations to diverge function.

### The Relationship with Functional Divergence and Structural Dynamics

**Figure 2:**
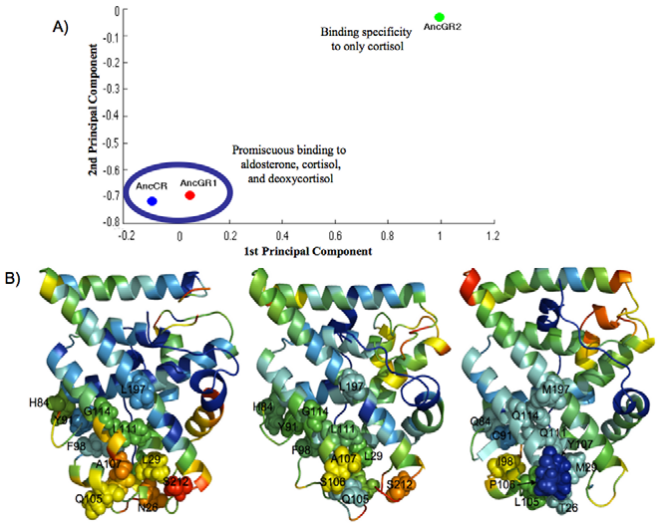
Plot and ribbon diagram of the dynamics of the three ancestral proteins characterized by slowest collective mode. (A) The first two principal components of AncCR, AncGR1 and AncGR2 plotted against each other. The principal components were found via a Singular Value Decomposition of the **G** matrix (See Methods). Higher order modes are mostly orthogonal or mixed and therefore not represented here. (B) 3D structures of AncCR, AncGR1 and AncGR2 colored by residue fluctuation. The critical mutations in AncCR and AncGR1 have greater flexibility and thus, higher binding promiscuity. AncGR2 has much lower flexibility in general amongst these residues and therefore more selective binding. The S212Δ mutation also rigidifies the lower loop at the bottom end of h10 by shortening the loop and removing degrees of freedom. This also alters the packing of h10 (the frontmost helix) and decreases flexibility.

We investigate the role of structural dynamics in functional divergence observed among the three ancestral steroid proteins. The extensive conformational sampling of our method enables us to capture the dynamics along with the most native-like structure (Fig. S4). We obtain the most collective modes of these three ancestral structures (i.e. slowest fluctuation profiles) through principal component analysis of our restraint-free trajectories (See Method). We then form an Mx3N matrix where the M columns are the eigenvectors weighted by their eigenvalues, with each M column being a 3 column super-element composed from the slowest modes of AncCR, AncGR1 and AncGR2 and N being the number of C-α atoms. We chose to analyze the top 10 slowest modes and therefore there are 30 columns. By performing a singular value decomposition on this matrix, we measure how the most collective motions of these three ancestral proteins are distributed in dynamic space. Interestingly, as shown in Fig. 2A, AncCR and AncGR1 are much closer and distinctively separated in dynamic space from the functionally divergent ancestor of the human glucocorticoid receptor, AncGR2. Clustering in dynamics space is significant because it shows that these structurally similar but functionally unique proteins differ in functionally governing dynamics, as observed in previous studies [42], [54]–[56]. Moreover, previous studies indicate that functionally critical mutations alter modes that characterize biologically functional motion, while random sequence variations typically have non-statistically significant impact on those modes [57]. These findings indeed suggest that the governing functional dynamics is encoded within the structure and that only critical mutations lead to a shift in collective motion and therefore in binding selectivity as well [55], [58].

Fig. 2B presents the color coded ribbon diagrams of these three ancestral proteins with respect to their functionally related collective fluctuation (obtained by PCA) profiles within a spectrum of red to blue, where rigid regions are denoted by blue/green and flexible regions are denoted with red/orange. Experimentally determined function altering mutations are highlighted in the sphere representation. Strikingly, residues in and near the functional site (i.e. binding site) are much more flexible for the two promiscuous enzymes (AncGR1 and AncCR) whereas the human ancestor AncGR2, which has affinity only to cortisol, has very rigid functional site residues. The new view of proteins states that, rather than a single structure with induced binding, proteins interconvert between bound and unbound conformations in the native ensemble. Thus, promiscuous binding proteins utilize greater flexibility to interconvert between a greater number of conformations in the native ensemble as compared to specific binding proteins. Therefore, our dynamic analysis agrees with the new view that while the promiscuous ancestors are more flexible around the functional site, the functional site rigidifies as Nature biases towards binding only a single ligand with greater affinity [1].

### Identification of Function Altering Mutations through Structural Dynamics

Upon confirmation that dynamics can indeed distinguish functional divergence, the next question is whether dynamics can indicate which residues in the protein are critical to diverging function. We investigate whether we can distinguish the mutations, including function altering and permissive (i.e. compensatory), that cause AncCR/GR1 to shift function to specifically bind cortisol as AncGR2 does, and also those that reverse the function of AncGR2 to promiscuously bind in the same way as AncCR/AncGR1.

**Figure 3:**
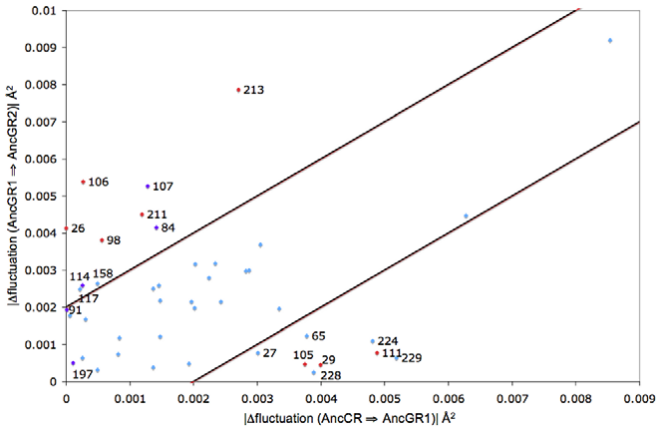
The change in fluctuation along the most collective mode between AncCR, AncGR1 and AncGR2. The X, Y, Z, and Y27R mutation groups necessary to alter function toward cortisol binding specificity are noted in red, and those permissive W mutations necessary to reverse function and recover promiscuous binding are noted in purple. A cutoff of ±0.002 Å^2^ is applied to differentiate mutations critical to altering dynamics as also used in Fig. 4. The upper left region of the graph indicates mutations that most alter dynamics when comparing the function-altering mutation from AncGR1 (binding promiscuity) to AncGR2 (binding specificity to cortisol) whereas the lower right region of the plot indicates mutations that most alter dynamics when comparing AncCR and AncGR1, which do not diverge functionally.

To identify the critical residues for swapping function, we analyze how the fluctuation profile changes over these three successive ancestral proteins. Thus, using their most collective fluctuation profile (i.e the slowest mode obtained by PCA), we compute the net change in fluctuation from AncCR to AncGR1 and AncGR1 to AncGR2 and show them in a 2-D plot to distinguish the mutations that have a higher impact on the change in dynamics between AncGR2 and AncGR1 compared to those mutations affecting the change in dynamics between AncGR1 and AncCR (Fig. 3). The upper left region of the graph in Fig. 3 indicates mutations that most alter dynamics when comparing the function-altering mutation from AncGR1 (binding promiscuity) to AncGR2 (binding specificity to cortisol) whereas the lower right region of the plot indicates mutations that most alter dynamics when comparing AncCR and AncGR1, which do not diverge functionally. The central region of the graph (between the parallel cutoff lines) contains those mutations that do not alter the dynamics in a significantly different manner between successive homologs. Interestingly, most of the function altering mutation sites such as 106, 212 (shown as 211 and 213 due to deletion) and most of the W mutations (mutations necessary for backward evolution, *e.g.* altering AncGR2 to become promiscuous) are in the upper left region. Permissive mutations 27, 29, 105, and the mutations in the activation function helix are in the lower right region of the plot. 111, a critical mutation for changing the specificity to cortisol only, is also in the lower right region. However, experimental analysis showed that the 111 mutation alone does not alter function in any appreciable manner. Thus, we propose it is only after permissive mutations alter the dynamics at site 111 can the necessary critical mutation at site 111 have a function altering effect. Additionally, certain mutations such as 214 and 173 both show large dynamic transitions. Mutation 214 is associated with the loop region that contains the critical mutation S212Δ, and it is in at the edge of a loop region. It undergoes transitions between being at the end of the h10 helix to being in the loop. The change in dynamics can be associated with the S212Δ mutation to identify the loop as a critical region. The 173 mutation is in a region that was not able to be crystallized in the experimental AncCR structure. Though the REMD simulations were determined to have converged, there is a possibility of some influence near site 173 due to the loop having to be built into the structure prior to REMD simulation. However, we expect that the shift in dynamics at mutation 173 may be correlated with movement of helix h10, and is therefore potentially significant.

**Figure 4:**
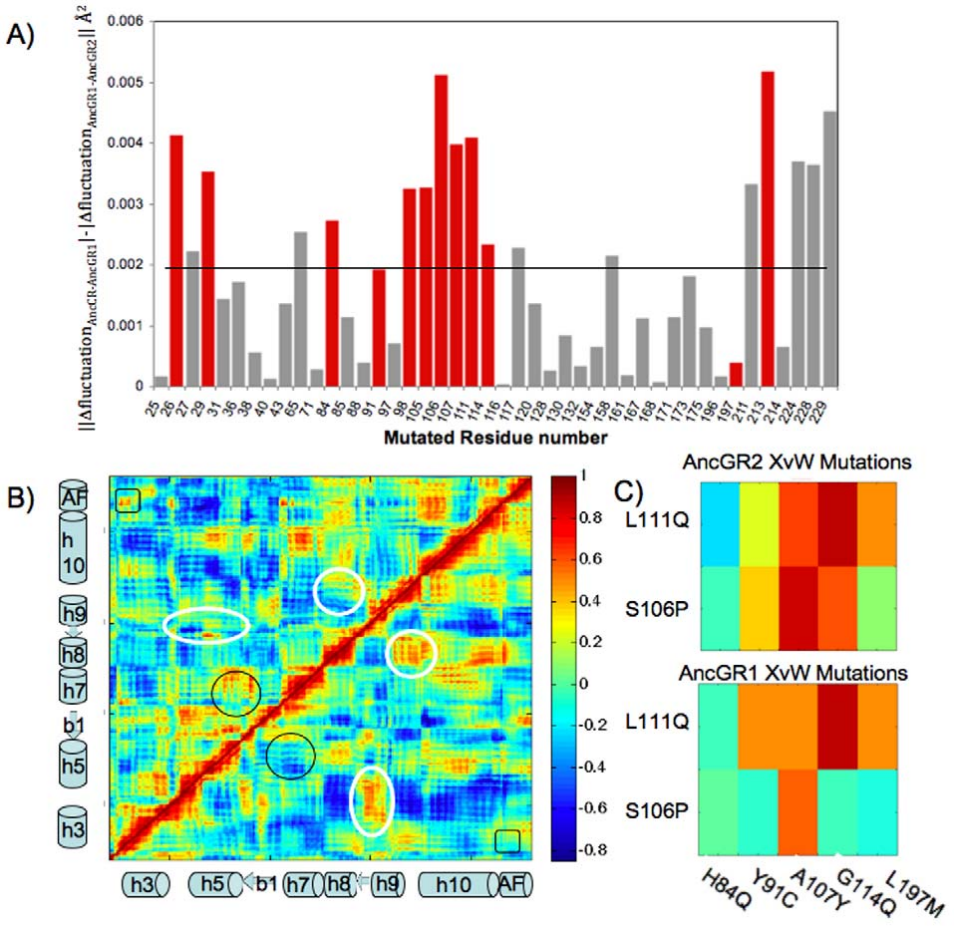
The change in net fluctuations and correlations of the mutated residues for successive evolution of MR to GR proteins. (A) The change in net fluctuation between successive ancestral proteins, AncCR, AncGR1 and AncGR2 for mutated residues. Those residues identified as critical to alter-function are noted in red. The activation-function (AF) helix contains mutations 224 and 229. A cutoff (solid line) results in all critical mutations identified except for Y91C and L197M. Y27R is noted as critical to function but sites 65, 117, and 158 are false positives. (B) The cross correlation map with AncGR2 on the upper left and AncGR1 on the lower right. Circled in black are changes in the cross correlation associated with critical residues near the binding pocket. Squared in black are the changes in cross correlation due to critical mutation N26T forming a hydrogen bond with the AF-helix. Circles in white are additional changes in cross correlation not associated with critical mutations. (C) The cross correlations between the X and W mutations. The correlation between X and W mutations is higher for AncGR2, whereas AncGR1 X functional mutations are uncorrelated, increasing the flexibility in the binding pocket and allowing for promiscuous binding.

We also obtain the net absolute change in the successive Δr^2^ fluctuation profiles along the slowest mode using the formulation ∥Δfluctuation_AncCR-AncGR1_|–|Δfluctuation_AncGR1-AncGR2_∥ for mutated residues based the alignment of AncCR and AncGR2 (Fig. 4A) and predict those residues with a net |ΔΔfluctuation|>0.002 Å^2^ to be critical. The forward mutations required to shift function to cortisol specificity are N26T, L29M, F98I, Q105L, S106P, L111Q, and S212Δ, and all of these are captured as critical as they are above the cutoff. The reverse mutations required to shift function from cortisol specific to promiscuous binding are H84Q, Y91C, A107Y, G114Q, and L197M. With the chosen cutoff, the identified permissive mutations are H84Q, A107Y, and G114Q, with Y91C only slightly below the cutoff. Interestingly, A107Y is the only W mutation that by itself partially recovered the promiscuous binding function [33] and it shows a high |ΔΔfluctuation| in our plot. We also find eight other mutated residues above the cutoff. Three of those are false positives I65L, Q117K and M158I. Each of these mutations occurred between AncCR and AncGR1, prior to a shift in function. Among mutations identified is Y27R, which is not explicitly in the X, Y, or Z set, yet it is highly conserved in the GR family and is an experimentally determined permissive mutation critical for GR function [34]. The three mutations at the activation function helix are also identified as critical. The other mutation above the cutoff is 211, which is correlated with S212Δ. Overall, our dynamic method identifies all mutations that are necessary for the evolution of GR function. We also distinguish three of the five mutations necessary for reversal of evolution (e.g. permissive mutations to AncGR2 which are necessary to recover the promiscuous binding of AncCR/AncGR1). Interestingly, many of the identified critical mutations such as N26T, H84Q, Y91C, F98I, Q105L, and S212Δ, are not interacting with the ligand, but rather are distant from the binding pocket (i.e. >5 Å from any atom in the ligand). Additionally, the high |ΔΔfluctuation| at the C-terminus is associated with the activation-function (AF) helix, which does not contain critical mutations but its dynamics is critical to function.

We also investigate the pairwise cross correlations of AncGR1 and AncGR2 (Fig. 4B). Interestingly, comparing the cross correlations reveals differences along the regions containing critical mutations. The cross-correlations between helix h5 (containing the critical mutation H84Q) and helix h7 (containing the critical mutations: Q105L, S106P, A107Y, L111Q, G114Q) become highly positively correlated in AncGR2 whereas there is no correlation in AncGR1. Analysis of hydrogen bonds [53] in predicted structures showed that additional hydrogen bonds are found between the β-sheet b1 and helices h5 and h7, indicating the observed increased correlation in AncGR2 is likely due to the repacking of helices h5 and h7 after mutation which incorporates/creates these new hydrogen bonds. Moreover, we also observe increased positive correlations between the AF-helix and helices h3 and h10 in AncGR2. These regions contain multiple permissive mutations (N26T, L29M, L197M, S212Δ) and thus, the change in correlations relate to the change in the stability of the AF helix caused by these permissive mutations necessary to alter function [34]. Furthermore, in Fig. 4C we compare the cross correlations of the most critical mutation for swapping the function to GR (X mutations) and the permissive mutations necessary to reverse the function to MR (W mutations) between AncGR1 and AncGR2. In AncGR2 these mutations are significantly more correlated than in AncGR1. This indeed suggests that W mutations play a critical role for GR function from the dynamics-perspective and therefore, they also need to be reversed along with the X, Y, Z mutation to recover the MR function.

To test the robustness of our method in other proteins we repeated our method for benign and disease associated mutations [59]–[61] in the human ferritin protein [62] (Fig. S5). We observe that, indeed, benign and disease associated mutations are individually clustered together while separated from each other in dynamics space.

In summary, by comparative dynamics analysis among the three ancestral steroid hormone receptors we identify all functionally critical and permissive mutations necessary to evolve new function from the ancestral MR promiscuous binding proteins to the ancestral GR cortisol-specific binding proteins. We also identify 60% of the permissive mutations necessary to revert to ancestral function along with an additional functionally critical mutation. We observe significant loss of flexibility in key residues both near and distant from the binding pocket in the transition from promiscuous to specific binding. A loss in flexibilty agrees well with the new view of proteins being conformationally dynamic in which bound and unbound conformations are sampled within the native ensemble. Thus, proteins evolve not just through those mutations that alter function in the immediate sense, but also due to those mutations that are permissive and alter the dynamic space in which the protein exists, thereby giving the protein the potential to evolve new function.

## Methods

### Ancestral Protein Structure Prediction Based on Modern Homologs

**Figure 5:**
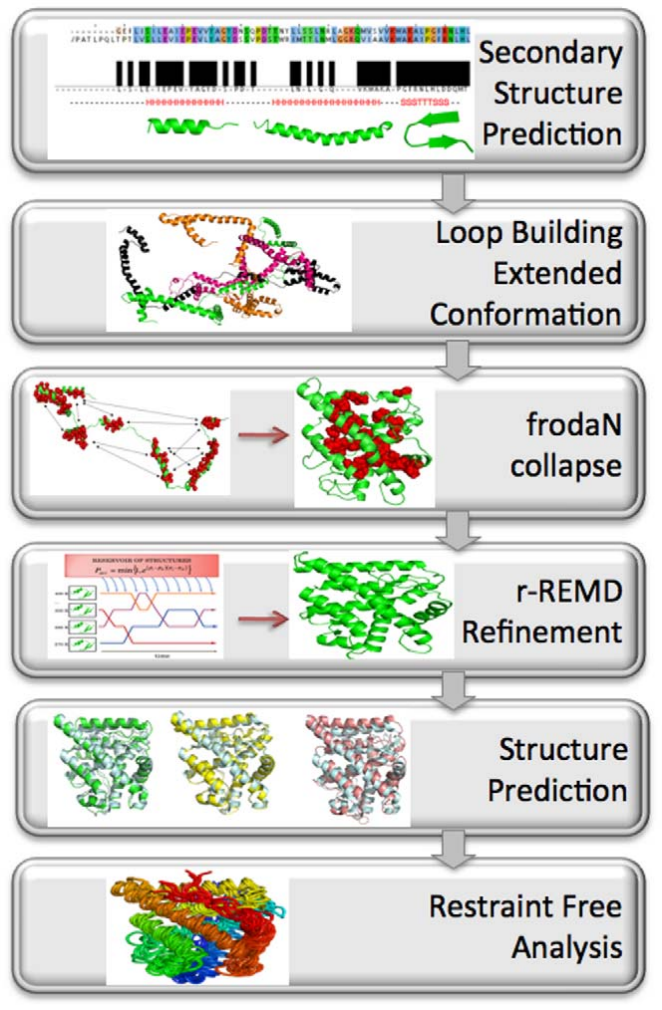
The secondary structure is predicted through multiple sequence alignment with modern day homologs. These secondary structural elements are then connected with loops in extended conformation to generate hundreds of conformations with high flexibility. Only a few are shown here. These structures all undergo a FRODA simulation which collapses them by adding attractive perturbations between all hydrophobic contact pairs (represented by arrows) into tightly packed structures with hydrophobic cores. A subset of hydrophobic residues are shown as spheres. After scoring, the collapsed structures they are ran in a restrained r-REMD simulation for 5 ns and then an unrestrained REMD simulation for 5 ns or until converged. The 3 ancestral structures are prediction to within 2.7 Å all atom RMSD of a similar experimentally determined structure. The final ensemble of restraint free generated structures are analyzed for dynamics using PCA.

We previously used the Zipping and Assembly Method with FRODA [ZAMF] [45]–[48], [63] on a set of test proteins to predict the 3D structure from their 1D amino acid sequence. Here, we slightly modify ZAMF for the prediction of ancestral protein structures, particularly the three ancestral steroid receptor proteins, the corticoid receptor [AncCR], the glucocorticoid/corticoid receptor [AncGR1], and the glucocorticoid receptor [AncGR2] [33], [34]. Since structure is more conserved than sequence [64]–[66], we incorporate structural data acquired from modern day homologues into our prediction method. The modified version of ZAMF as outlined in Fig. 5 includes several steps: (i) obtaining secondary structural motifs and common contacts based on modern homologs, (ii) generation of an unfolded ensemble, (iii) generation of compact-native like conformations using FRODA, and (iv) refinement by ZAMF. Overall, all these steps lead to an extensive search in conformational space, which comes with several advantages. First, we increased our prediction accuracy for native structures compared to the previous version of ZAMF. Second, we obtain converged dynamics trajectories through the refinement stage of ZAMF, which is used for dynamic evolution analysis of the ancient proteins. We summarize each step in our approach below.

#### I. Obtain secondary structural motifs and potential contact map of ancestral sequences

Usually, the first stage of ZAMF is to predict the secondary structural elements for shortened sequences, i.e., 8mers, 12mers, 16mers etc of the protein using an *ab initio* approach. However, here we use the SSPRED online server [67] to confirm likely secondary structural elements by examining the secondary structure of modern day homologs such as mouse, human, and rat steroid receptor proteins [68]–[71] and aligning with the ancestral sequences. We choose the predicted secondary structural motifs such that they agree with the secondary structural motifs of modern day homologs at the regions with high sequence similarity. Furthermore, the information gleaned from the sequence alignment of the modern day homologs is also coupled with analysis of the 3D structure of the modern day homologs in order to generate a contact map for the target ancestral protein in question. For example, if segment of modern-day homolog 10–15 and 20–26 have identical residues with those of ancient sequence and there is a contact between 10 and 20, we use the contact 10, 20. In order to translate these contact maps between each other, we take into account insertions, deletions and differences in numbering from the sequence alignment. Finally, the consensus contacts across all maps (i.e. contacts overlap in all modern day homologs) are taken as the contact map for the ancestral proteins. The contact map includes both residue-residue distance contacts and also dihedral angle variations. This contact map is later used to couple with FRODA [47], [72] during simulations that collapse the assembled secondary structural motifs into folded units.

#### II. Generation of unfolded assembled secondary structural motifs

The individual secondary structure elements are connected by building loops in extended conformation between secondary structures adjacent in sequence. We use a Monte Carlo technique in ZAMF [63] to build these loops and generate hundreds of unique conformations each with maximized radii of gyration, as shown in Fig. 4. Using many initial structures has the advantage of unbiasing the results from any individual initial structure.

#### III. Generation of collapsed folded conformations using geometric constraint-based FRODA

Each of these unique, “open” structures is then run in a FRODA simulation that enforces hydrophobic collapse through attractive perturbations between specific hydrophobic residue pairs in the previously mentioned contact map. No hydrophobic residues within loops are chosen and contacts within the same secondary structural motif are not considered a contact pair. During the simulation each of the residue-residue contacts are perturbed together if their separation distance in >7.0 Å. The run is prematurely ended if all the contacts from the contact list are found to be within a 7.0 Å cutoff distance at any time during the simulation. An additional hydrophobic collapse of all hydrophobic residues is done via a Monte Carlo accept/reject method with Boltzmann weighting between subsequent snapshots based on the difference of radius of gyration of hydrophobic residues. Other parameters of the FRODA simulation, such as momentum run-on between subsequent steps, remain the same as outlined in previous work [45].

The final collapsed structures from the FRODA simulations are then clustered into representative structures using a *k*-means clustering algorithm based on a 1.0 Å RMSD between atomic positions. These representative structures are scored and sorted based on both the radius of gyration of hydrophobic residues and also the number of hydrophobic contacts (<7.0 Å) (Fig. S6).

#### IV. Refinement and selection of the most native-like folded structure using ZAMF

We then move on to the refinement stage of ZAMF. The refinement stage involves a reservoir REMD (r-REMD) [73] step to both determine the most native conformation and also to further refine all conformations. The replicas and reservoir are filled with structures that are sorted according to the hydrophobic scoring function mentioned above. We then run multiple simulations where we narrow the conformational search space to avoid entrapment in local minima through residue-residue contact restraints based on the contact map of the ancestral protein. The local contacts are applied before the nonlocal ones to allow local refinement to occur before global refinement (tertiary structure). This approach is motivated by a hierarchical folding mechanism (search mechanism of ZAM). The restrained simulation is ran for 5 ns with replicas from 270K to 450K in the AMBER96 force field with generalized born implicit solvent model [74]. The residue-residue constraint is approximated to be at the center of mass of the residue and the force constant is 0.5 kcal/(mol Å^2^). After the restrained run, an unrestrained simulation with identical parameters is then run for at least 5 ns. After 5 ns, a convergence analysis is done, and if the protein is converged no further simulation is completed. If it is not, an additional 2 ns of simulation is run and convergence is checked. Continued 2 ns simulations are repeated until the protein has converged. The most dominant structure at the lowest replica is chosen as our prediction at the end of convergence. Our refinement protocol works well for ancestral sequences since their structure is close to modern day homologs whose structures are known. In other extreme cases where the starting initial model has lower resolution (i.e. 6–7 Å RMSD) from the original structure, our refinement protocol may fail and need additional alterations in order to reach to higher resolution structures.

Since we also generate an extensive amount of trajectory data, we use the unrestrained converged trajectories to analyze the dynamics of the ancestral structure as explained in detail below.

### Principal Component Analysis for Identifying Functionally Important Dynamics

Convergence is critical and, as such, a sample window of 1 ns is slid along the trajectory at 0.5 ns intervals and Principal Component Analysis is done. The PCA is done by first aligning and centering each snapshot of the trajectory to remove the translations and rotations, generating a matrix **X_n_** for each sampling window
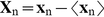
(1)where **x_n_** are 3N dimensional position vectors and the < > denote a time average for a specific sampling window. Then, the covariance matrix of that sampling window, **C_n,n_**, is calculated by
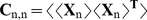
(2)From the covariance matrix, the matrix of eigenvectors (**V_n_**) and the matrix of eigenvalues (**Λ_n_**) are
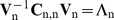
(3)The eigenvectors and eigenvalues are sorted in order of decreasing eigenvalue and only the top 30 are kept as, once converged, any higher order (faster fluctuation/smaller positional deviations) are not relevant in determining biologically relevant large scale motion of the protein [75]. The reduced set of principal components is then
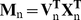
(4)The fluctuation profile along each mode is simply the Δr of each residue in that mode. By plotting these against each other, we confirm convergence when the Pearson correlation coefficient, *P_ij_*, of the trajectory for sampling window i (**X**_i_) and sampling window j (**X**_j_) is >0.8
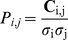
(5)σ_i_ and σ_j_ are the standard deviations of their trajectories. If the run has not converged it is continued until convergence is confirmed over a 3 ns window (Fig. S3). Using the Saguaro high performance computer at Arizona State University, a 250 residue protein with 40 temperature replicas (1 logical core per replica) finishes just under 300 ps/day. The most native like structures are assumed to be those that dominate the lowest temperature replica, while those in higher temperature replicas are dismissed.

After confirming convergence, in order to obtain the dynamics difference between the most collective motions (i.e. slowest frequency fluctuation profiles) of these three ancestral structures we apply the Singular Value Decomposition (SVD) technique to the matrix of dynamics profiles, **G** (i.e. the dynamics profile of each protein will be the column in the matrix, and each super-element, *ik* corresponds the X, Y, and Z fluctuations of the *k* ^th^ residue in the sequence of protein *i*).
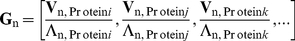
(6)G matrix includes most collective modes of (i.e. global motion) individual proteins that we obtained separately from REMD trajectories. With construction of the G matrix our goal is to cluster the proteins with similar global motion. Since global dynamics (i.e. most spatially extensive collective mode) is most related to the function, proteins with similar global dynamics should cluster together and execute similar function. In order to do clustering we perform an SVD on G matrix
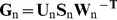
(7)The first through *n*th values in each column of **W** can be plotted against each other to visualize the dynamic space occupied by each protein.

## Supporting Information

Figure S1 The RMSD versus time plot for experimental structures of AncCR and AncGR2.
(PDF)

Figure S2 The Mean Square Displacement between our predicted structures for AncCR-AncGR1 (blue), AncCR-AncGR2 (green), and AncGR1-AncGR2 (red).
(PDF)

Figure S3 The plot of most collective mean square fluctuation of different sliding windows.
(PDF)

Figure S4 The dynamics of the experimental AncCR, AncGR1, and AncGR2 structures plotted in a reduced subspace.
(PDF)

Figure S5 Plot and ribbon diagram of the dynamics of the single mutation variant of human ferritin protein characterized by the slowest collective mode.
(PDF)

Figure S6 Radius of gyration of the hydrophobic residues versus the RMSD from the experimentally determined structure of AncCR for a single FRODA run.
(PDF)

Table S1 RMSD from Native Before and After REMD Simulation.
(PDF)
