# Supplementary material for: Collective Dynamics Differentiates Functional Divergence in Protein Evolution
Source: PLoS Comput Biol. 2012 Mar 29;8(3):e1002428. doi: 10.1371/journal.pcbi.1002428 (PMC3315450; doi:10.1371/journal.pcbi.1002428)
Supplement: Figure S1 — The RMSD versus time plot for experimental structures of AncCR and AncGR2. (PDF) [file pcbi.1002428.s001.pdf]

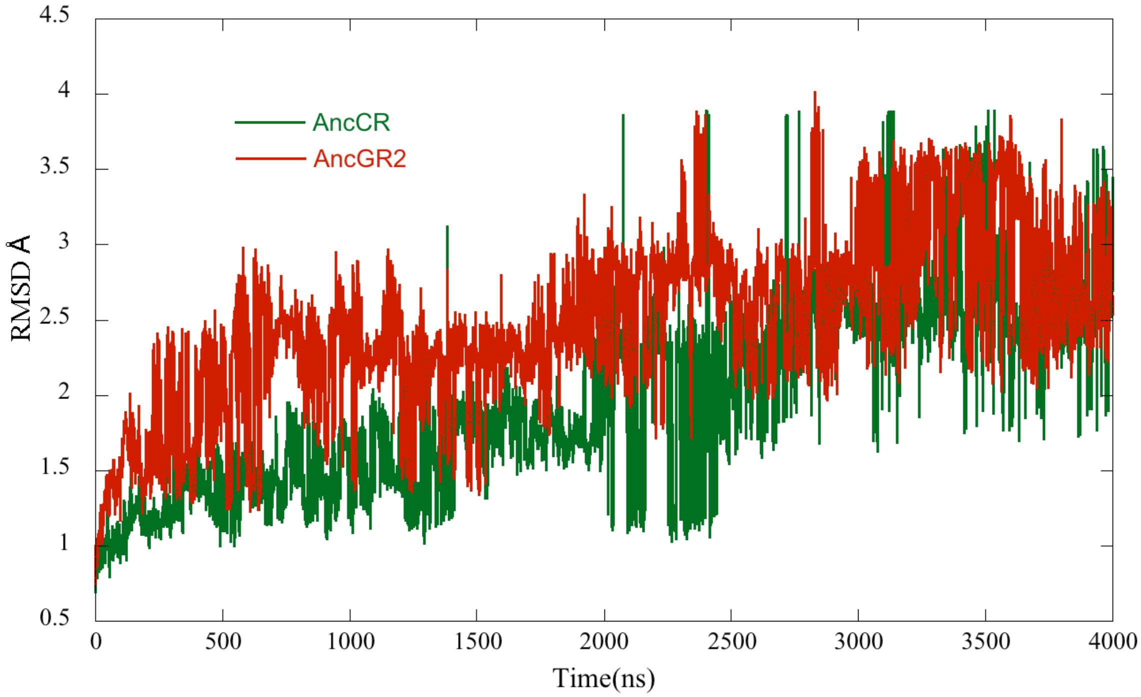

Fig. S1: The rmsd versus time plot for experimental structures of AncCR and AncGR2. Two separate 4ns REMD simulations in the Amber 96ff with generalized born implicit solvent model and 40 logarithmically spaced temperature replicas ranging from 270-450 K were run with the experimentally determined native structure of AncCR and AncGR2 as initial structures. We obtained a total of 160 ns simulation time for each protein. The backbone rmsd from the experimental structures in the lowest temperature replica was computed. It converges to ~2.5Å backbone rmsd for both AncCR (green) and AncGR2 (red). Thus, as these proteins are stable in the Amber 96ff, it is an acceptable choice of force field for this study.
