# Supplementary material for: Collective Dynamics Differentiates Functional Divergence in Protein Evolution
Source: PLoS Comput Biol. 2012 Mar 29;8(3):e1002428. doi: 10.1371/journal.pcbi.1002428 (PMC3315450; doi:10.1371/journal.pcbi.1002428)
Supplement: Figure S2 — The Mean Square Displacement between our predicted structures for AncCR-AncGR1 (blue), AncCR-AncGR2 (green), and AncGR1-AncGR2 (red). (PDF) [file pcbi.1002428.s002.pdf]

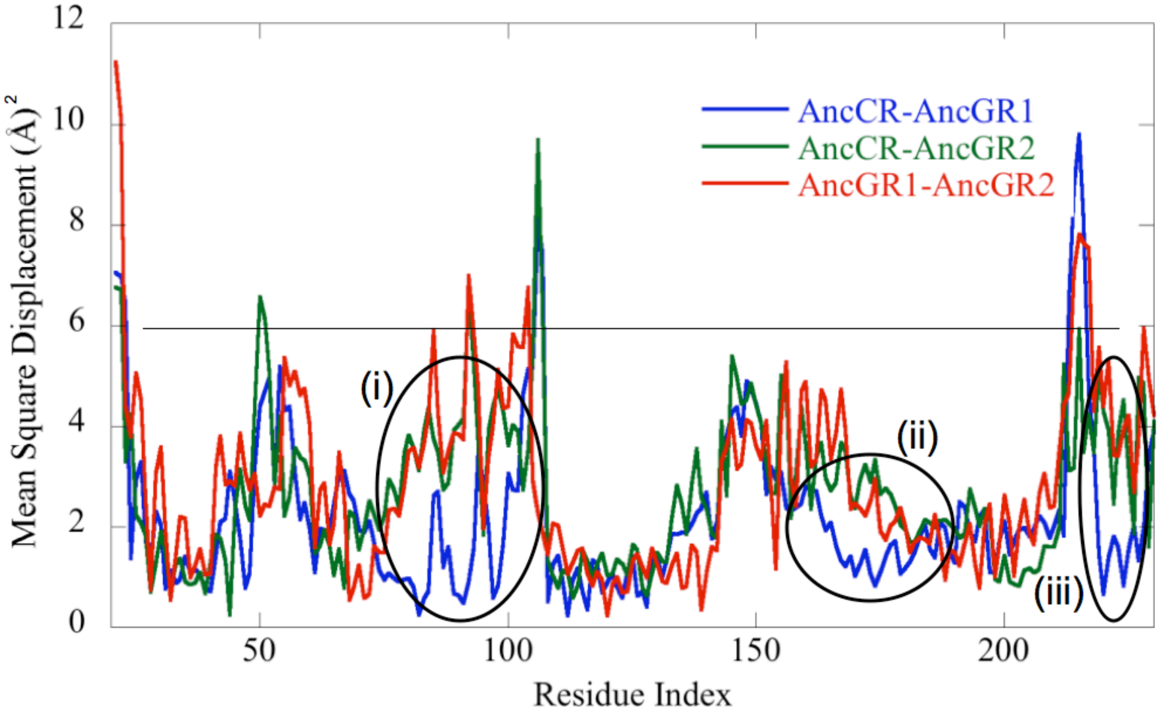

Fig. S2: The Mean Square Displacement between our predicted structures for AncCR-AncGR1 (blue), AncCR-AncGR2 (green), and AncGR1-AncGR2 (red). The three large structural changes between successive generations are associated with the shift in; (i) β-sheet b1 and helices 5, 6 and 7; (ii) helix 9 and the top of helix 10; (iii) the bottom loop of helix 10 and the activation function helix.
