# Supplementary material for: Collective Dynamics Differentiates Functional Divergence in Protein Evolution
Source: PLoS Comput Biol. 2012 Mar 29;8(3):e1002428. doi: 10.1371/journal.pcbi.1002428 (PMC3315450; doi:10.1371/journal.pcbi.1002428)
Supplement: Figure S3 — The plot of most collective mean square fluctuation of different sliding windows. (PDF) [file pcbi.1002428.s003.pdf]

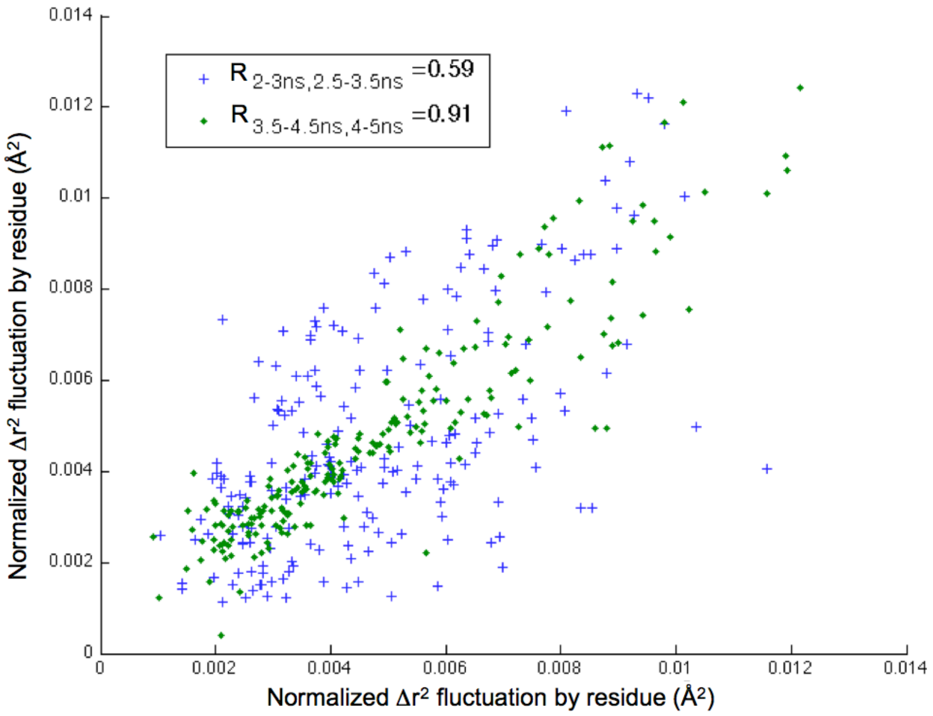

Fig. S3: The plot of most collective mean square fluctuation of different sliding windows. sIn order to check convergence we plot the most collective mean square residue fluctuation obtained from the trajectory windows of 2.0-3.0 ns against that of 2.5-3.5 ns (blue). Likewise, we repeat this analysis for the sliding time windows of 3.5-4.5 ns and 4.0-5.0 ns (green). The trajectory has not converged by 3.5ns, as the Pearson correlation coefficient between the 2.0-3.0ns and 2.5-3.5ns windows is R=0.59. After 5ns the trajectory has converged, as the Pearson correlation coefficient between windows 3.5-4.5ns and 4.0-5.0ns windows is R=0.91.
