# Supplementary material for: Collective Dynamics Differentiates Functional Divergence in Protein Evolution
Source: PLoS Comput Biol. 2012 Mar 29;8(3):e1002428. doi: 10.1371/journal.pcbi.1002428 (PMC3315450; doi:10.1371/journal.pcbi.1002428)
Supplement: Figure S4 — The dynamics of the experimental AncCR, AncGR1, and AncGR2 structures plotted in a reduced subspace. (PDF) [file pcbi.1002428.s004.pdf]

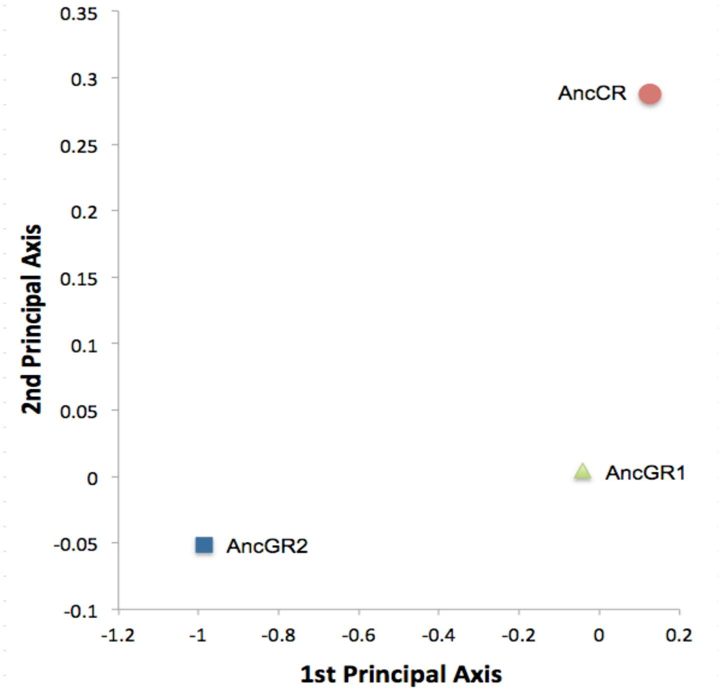

Fig. S4: The dynamics of the experimental AncCR, AncGR1, and AncGR2 structures plotted in a reduced subspace. While there is a clear differentiation between AncGR2 and AncCR/AncGR1 along the first principal axis, this does not hold for the second principal axis. Therefore we conclude that the ZAMF procedure allows for a more efficient sampling of dynamic space due to the larger number of initial conformations used.
