# Supplementary material for: Collective Dynamics Differentiates Functional Divergence in Protein Evolution
Source: PLoS Comput Biol. 2012 Mar 29;8(3):e1002428. doi: 10.1371/journal.pcbi.1002428 (PMC3315450; doi:10.1371/journal.pcbi.1002428)
Supplement: Figure S5 — Plot and ribbon diagram of the dynamics of the single mutation variant of human ferritin protein characterized by the slowest collective mode. (PDF) [file pcbi.1002428.s005.pdf]

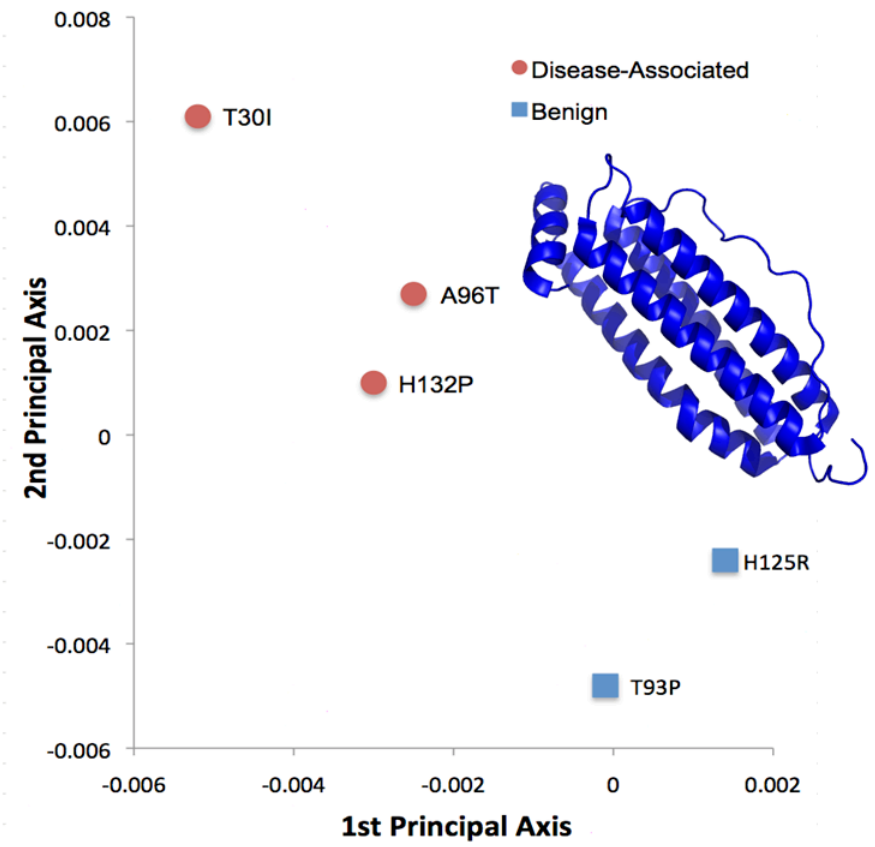

Fig. S5: Plot and ribbon diagram of the dynamics of the single mutation variant of human ferritin protein characterized by the slowest collective mode. The first two principal components of benign and damaging mutations (i.e. leading to functional loss) plotted against each other and they are separated based on dynamics space. The principal components were found via a Singular Value Decomposition of the **G** matrix.
