# Supplementary material for: Collective Dynamics Differentiates Functional Divergence in Protein Evolution
Source: PLoS Comput Biol. 2012 Mar 29;8(3):e1002428. doi: 10.1371/journal.pcbi.1002428 (PMC3315450; doi:10.1371/journal.pcbi.1002428)
Supplement: Figure S6 — Radius of gyration of the hydrophobic residues versus the RMSD from the experimentally determined structure of AncCR for a single FRODA run. (PDF) [file pcbi.1002428.s006.pdf]

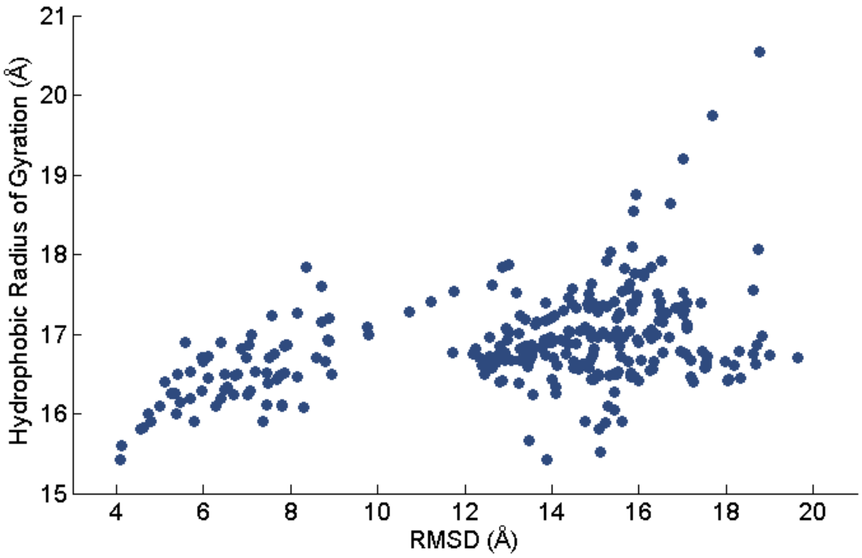

Fig. S6: Radius of gyration of the hydrophobic residues versus the rmsd from the experimentally determined structure of AncCR for a single FRODA run. By using the hydrophobic radius of gyration we select against non-native like structures and only seed the ensuing REMD simulations with collapsed structures, many of which are native-like.
