# Supplementary material for: Collective Dynamics Differentiates Functional Divergence in Protein Evolution
Source: PLoS Comput Biol. 2012 Mar 29;8(3):e1002428. doi: 10.1371/journal.pcbi.1002428 (PMC3315450; doi:10.1371/journal.pcbi.1002428)
Supplement: Table S1 — RMSD from Native Before and After REMD Simulation. (PDF) [file pcbi.1002428.s007.pdf]

Table S1: RMSD from Native Before and After REMD Simulation

RMSD from Experimentally Determined Structure (Å)			
	**FRODA Ensemble**	**FRODA Best Structure**	**REMD Best Structure**
**AncCR**	**14.33**	**2.8**	**2.5**
**AncGR1**	**16.52**	**4.1**	**2.9**
**AncGR2**	**9.00**	**3.9**	**2.8**

Table S1 indicates the rmsd from native for multiple steps in the structure prediction algorithm. The geometric constraint based step generates a large number of structures, among which only a few are native like. This can be seen from the high rmsd average of the ensemble. By seeding the REMD simulation with many structures from the FRODA ensemble we are able to isolate the most native-like structures from the REMD simulation and refined it to a better RMSD.
